# Conceiving Particles as Undulating Granular Systems Allows Fundamentally Realist Interpretation of Quantum Mechanics

**DOI:** 10.3390/e23101338

**Published:** 2021-10-14

**Authors:** Stéphane Avner

**Affiliations:** CNRS, Univ. Rennes, IGDR—UMR 6290, F-35000 Rennes, France; stephane.avner@univ-rennes1.fr

**Keywords:** quantum mechanics interpretation, classical physics fundamental principles, granular systems, dynamical systems

## Abstract

The strange behavior of subatomic particles is described by quantum theory, whose standard interpretation rejected some fundamental principles of classical physics such as causality, objectivity, locality, realism and determinism. Recently, a granular relativistic electrodynamical model of the electron could capture the measured values of its observables and predict its mass from the stability of its substructure. The model involves numerous subparticles that constitute some tight nucleus and loosely bound envelope allegedly forming real waves. The present study examines whether such a substructure and associated dynamics allow fundamentally realist interpretations of emblematic quantum phenomena, properties and principles, such as wave-particle duality, loss of objectivity, quantization, simultaneous multipath exploration, collapse of wavepacket, measurement problem, and entanglement. Drawing inspiration from non-linear dynamical systems, subparticles would involve realist hidden variables while high-level observables would not generally be determined, as particles would generally be in unstable states before measurements. Quantum mechanics would constitute a high-level probabilistic description emerging from an underlying causal, objective, local, albeit contextual and unpredictable reality. Altogether, by conceiving particles as granular systems composed of numerous extremely sensitive fluctuating subcorpuscles, this study proposes the possible existence of a local fundamentally realist interpretation of quantum mechanics.

## 1. Introduction

In a vulgarization article, Wiseman introduced the issue of local realism as follows [1]: “The world is made of real stuff, existing in space and changing only through local interactions—this local-realism hypothesis is about the most intuitive scientific postulate imaginable. But quantum mechanics implies that it is false”.

For most physicists who accept the precepts of the standard Copenhagen interpretation of quantum mechanics [2], physics needs not seek to apprehend reality, but should only be concerned with predicting the outcomes of measurements [3]. However, *realist physicists* believe they should primarily try to understand, in the essential meaning of that word, i.e., to uncover the world’s underlying nature: the well-defined properties of its objects and environment, and the laws and principles that govern them. Smolin emphasizes that understanding reality remains the *ultimate mystery* and that the very existence of reality and objectivity in the whole of science are at stake [4]. The solutions that will be eventually established may have consequences on our general attitude towards ideas such as relativism, according to which truth would be only relative and every explanation interchangeable. Our conceptions regarding these issues might ultimately depend on our scientific description of the infinitely small, since nature at every level of observation would be determined by the laws applying at a lower level.

The behavior of subatomic particles is currently described by quantum theory [5], which has effectively abandoned some long-established fundamental principles of classical physics, such as *causality* (every effect has a cause and does not occur at random), *objectivity* (every object of study effectively exists and behaves independently of the existence of an observer), *locality* (particles and fields interact locally and propagate no faster than light velocity), *physical realism* (in Einstein’s view, objects possess definite properties), or *determinism* (outcomes of measurements are determined by pre-existing properties of objects), even though those principles had long provided invaluable guidance to physicists [6]. Noteworthy, the concept of *‘reality’* has different meanings in physics. By *reality*, Einstein et al. [7] meant that particles possessed definite properties. Accordingly, Born wrote: “We regard waves on a lake as real, though they are nothing material but only a certain shape of the lake surface. The justification is that they can be characterized by certain invariant quantities” [8]. Incidentally, de Broglie proposed a more geometrically oriented definition: “Abstract presentations have no physical reality. Only the movement of elements localized in space, in the course of time, has physical reality” [9]. Nowadays however, these fundamental classical principles seem incompatible with the strange behavior of particles observed in various quantum phenomena. For instance, particles appear to exhibit two different behaviors, corpuscular and wave-like, each being irreducible to the other, depending on the experiment being performed, thus challenging the principle of objectivity and leading to the comprehensive but incomprehensible concept of *wave-particle duality* [2], as Bohr’s complementarity could be seen as acceptance of contradiction [10].

The quantum mechanical description in terms of operators and wavefunctions is so abstract that an interpretation is needed to specify what is actually being described [11]. Its standard interpretation advocates that observed phenomena reveal a reality so remote from standard perception that it is unintelligible for the human mind [12], that particles cannot be conceived in geometrical terms, and that the only possible description must be probabilistic in essence. Its standard interpretation is (*i*) *non-causal*, as electrons in the atom for instance are thought to keep appearing and disappearing at every position with given probabilities, instead of following determined trajectories, (*ii*) *non-objective*, as particles exhibit properties that depend on the kind of measurement being performed, (*iii*) *non-deterministic*, as quantum systems exhibit random measurement outcomes (*stochasticity*), and either (*iv*) *non-local*, postulating the existence of non-separable distant systems or allowing superluminal (faster than light) interactions, or (*v*) *non-realist*, as particles do not seem to possess definite properties. For Bohr [13] and those committed to the Copenhagen interpretation who advocate the intrinsically probabilistic nature of atomic and particle physics, quantum theory is the ultimate description of nature beneath Compton scale, in the sense that it cannot be refined. According to Sulis, “quantum mechanics is considered complete epistemologically [14]; whether it is complete ontologically is no longer a criterion” [15]. 

Quantum mechanics however has its own unresolved issues. First, the macroscopic and microscopic physics paradigms are incompatible [16]. The standard interpretation “denies the application of the concept of reality to atoms, electrons, fields, etc. […] But where is the border between these two domains?” [6]. Fundamentally, should not all phenomena be governed by only one kind of logic [17]? Second, it is unclear when to apply Schrodinger’s equation, which describes the deterministic linear evolution of the wavefunction, and when to invoke von Neumann’s non-linear *collapse of the wave packet* [18], in which the wavefunction jumps into a pure state or eigenstate. These correspond effectively to two different dynamical evolutions of quantum systems. Specifically, the issue arises because an objective criterion, specifying how the particle switches from one dynamical evolution to the other, is currently lacking [19,20]. Next, the observer seems to play an active role in the theory: the collapse is triggered by the observer’s measurement [21], apparently challenging the principle of objectivity. For Popper, an objective theory requires that the formalism does not rely on the notion of observer [22]. According to Born [6], “quantum mechanics has destroyed the distinction between the object and subject”, as the measuring apparatus is now part of the description. Reacting to considerations by which even consciousness should be included in the description of measurements [23], Einstein notoriously pondered: “Is the moon there when nobody looks?” [24,25], while Allori [26] pointed out that “an appeal to consciousness is equivalent to the rejection of the completeness of physics”. Further, the *measurement problem* [27] questions why a quantum system always ends up in a quantized eigenstate upon measurement, while the system is thought to be in a *superposed state* in general. The issue of superposition is illustrated by Schrödinger’s cat paradox [21], which compares the quantum description of a particle to a cat that would be simultaneously both living and dead. In their thought experiment, Einstein, Podolsky and Rosen [7] further objected that, in the absence of definite properties, the existence of correlations between entangled particles would imply that quantum theory contradicts locality. The violation of Bell’s inequalities [28] by experiments (e.g., [29,30,31]) implies that, if we choose to conserve locality, a solution compatible with the predictions of quantum mechanics must be intrinsically *contextual* [32,33,34], unpredictable [35], and present *non-Kolmogorovian probabilities* [36,37]. The standard interpretation of quantum mechanics also rejects reality and determinism through Heisenberg’s *uncertainty principle* [38], which asserts that noncommuting observables of a particle cannot both be known with exactitude simultaneously. If the purpose of physics is to understand phenomena, rather than provide mere probabilistic predictions, physicists should feel unsatisfied with the current interpretation of quantum theory.

Despite the existence of several disputed mathematical impossibility proofs or so-called *no-go theorems* [18,39,40,41] excluding the possible existence of an underlying *hidden variable theory* [18] that would determine in principle the outcome of individual measurements [20], other interpretations of quantum mechanics were proposed [26,42]. Everett’s many-worlds interpretation [43] for instance suggests that all possible states exist in parallel worlds. However, Bell pointed out that it was an extreme case of lack of economy of concepts [26,44]. Ballentine [45] considers the quantum description as applying to an ensemble of similarly prepared states. Bub [46] advocates an information-theoretic interpretation in which quantum mechanics represents and manipulates information. The *de Broglie–Bohm pilot wave interpretation* [47,48,49] proposes instead that a corpuscle be surrounded by an extensible wave, which would guide the corpuscle through its environment. Note that according to Bush, “the physical nature of the guiding wave field remains unclear” [50]. Another interpretation due to de Broglie [51,52] is the so-called double solution, which assumed that two distinct waves actually exist, both satisfying Schrödinger’s equation, one describing the dynamics of the guiding wave, and the other consisting in a densely contracted wave constituting the corpuscle. Hydrodynamical interpretations of quantum mechanics have also been proposed [53,54,55], but the substance composing the fluid was not specified. An interpretation of quantum theory resting on a lower level of description is allegedly still lacking [4,56], although proponents of the Copenhagen interpretation claim it is unnecessary [20].

Recently, a causal, objective, local, and realist relativistic electrodynamical model of the electron was proposed [57]. In this model, essentially intertwined charged subparticles revolve at light velocity in coplanar circular orbits, forming an envelope and nucleus. This corpuscular model provides a natural interpretation for every observable, and notably allows predicting electron mass and muon mass directly from an objective criterion: the stability of their substructure. In this theory, electrons are regarded as extended systems composed of numerous subparticles, whose dynamics could allegedly form periodic waves. The complex envelope dynamics would potentially exhibit stable states, like modes of a vibrating rope. The proposed substructure and associated dynamics present features that may be generalized to all subatomic particles. This model will be denoted the granular electron model in the remainder of this study. Its structure (made of numerous sub-elements subject to cross-interactions) and associated dynamics could be perceived as resembling those of Recurrent Neural Networks [58], which exhibit non-linear dynamics [59] converging towards a limited set of stable states called attractors. Likewise, the eigenstates of quantum mechanics could correspond to attractors of the system, suggesting how subatomic particles could exhibit *quantization*. Noteworthy, some macroscopic systems have also evidenced quantum-like properties [60,61,62], even if they may not be entirely comparable [63], and simulations in quantum optics [64] were realized without solving a wave equation [65] while still satisfying Einstein’s criterion of local causality.

In the present article, it is investigated whether such granular particle models could effectively exhibit phenomena and properties specific to quantum systems and be compatible with principles of quantum theory. Is it irrevocably impossible, as some founders of quantum mechanics (Bohr, Born, Heisenberg, Pauli, etc.) have professed, to conjecture a single interpretation preserving at least some of the aforementioned classical principles? Recall Sherlock Holmes saying in one of Conan Doyle’s novels: “Once you eliminate the impossible, whatever remains, however improbable, must be the truth”. Thus, would it be possible to conjecture an explanation of the quantum phenomenon, however improbable or tortuous, in causal, objective, local or realist terms, using the recent granular electron model as an inspiration? Could we form plausible hypotheses that would in effect allow reinterpreting observed quantum phenomena in fundamentally realist terms? All quantum properties and phenomena would need to be re-examined in the light of these hypotheses. Smolin [4] reckons that a realist interpretation should rest upon some *non-quantum* underlying theory to convey novel elements of reality. The aim would be to understand, i.e., to depict what could lie beneath quantum processes, by providing a finer description, possibly involving hidden variables lying at the level of some substructure [66] defined in terms of subparticles for instance. This prospect constitutes a novel perspective, possibly leading to a *complete and coherent* interpretation of quantum mechanics (an interpretation would be “complete, if and only if it provides a physical interpretation for every significant feature of the mathematical theory, and coherent, if and only if the interpretation for each feature fits naturally into a unified interpretation of the whole” [66]). Investigating this possibility is the purpose of the present article.

After briefly reviewing the properties of quantum systems that seem to be unintelligible in classical terms, and after enunciating key characteristics of the granular electron model, it will be examined whether novel hypotheses allow conjecturing coherent interpretations of emblematic quantum experiments (Young’s two-slits experiment, Mach–Zehnder interferometer experiment, one-dimensional potential well), quantum properties (wave-corpuscle duality, quantization, simultaneous multipath exploration, loss of objectivity, unpredictability, collapse of wavefunction, measurement problem, entanglement), and principles (complementarity, superposition, Heisenberg’s uncertainty principle, Pauli’s exclusion principle), in terms of systems of numerous subcorpuscles, while still remaining compatible with at least some of the fundamental principles of classical physics. The present proposal suggests the possible existence of a causal, objective, local, *fundamentally realist* (i.e., defined in terms of realist subprocesses but denying definite values for high-level observables in general—see below), albeit contextual and unpredictable interpretation of quantum phenomena, properties, and principles. A corpuscular worldview is introduced (*the granular interpretation of quantum mechanics*), which proposes that all particles and waves are ultimately reducible to subcorpuscles, and all quantum features eventually intelligible in terms of their properties and dynamics. Corpuscular and wave-like behaviors would be exhibited by the particle’s nucleus and envelope, respectively. The envelope would be conceived as a non-linear dynamical system possessing stable states, towards which some generally unstable dynamics would converge upon measurement. As *local realism* has been invalidated by the experimental violation of Bell’s inequalities, *high-level realism*, which advocates the existence of predetermined high-level properties, is rejected in order to conserve locality. In our conception, high-level observables would remain indefinite, as particles would generally be in unstable states before measurements, while underlying hidden variables would be determined, with subcorpuscles possessing definite positions and momenta and undergoing causal non-linear dynamics. Further, outcomes of measurements would be inherently contextual and unpredictable, as they would depend on the underlying positions and momenta of the subcorpuscles composing both the measured system and measuring apparatus.

All quantum phenomena seem to be compatible with our proposed granular interpretation. As the study aims at presenting a coherent and unified interpretation for most emblematic quantum properties, only concise propositions will be provided. Should our conjectures be experimentally verified, the description of nature beneath Compton scale would become compatible with most fundamental principles of classical physics, possibly allowing quantum mechanics to emerge from a causal objective theory such as *relativistic electrodynamics* [67]. As illustrated in the granular electron model, geometrically realist models could possibly be devised for all subatomic particles. The present interpretation would also provide new insight regarding the unification of the two apparently irreconcilable paradigms in physics: the macroscopic realist paradigm and microscopic quantum paradigm.

## 2. Classical Principles Apparently Cannot Account for Quantum Properties

So, what are those quantum properties that apparently disqualify the aforementioned fundamental classical principles? Here, we shall concentrate on a few emblematic properties, which appear to have no counterpart in classical physics and seem utterly unintelligible in classical terms: wave-corpuscle duality, quantization, exploration of all possible paths, loss of objectivity, wavefunction collapse, measurement problem, unpredictability, and entanglement.

In his thesis [68], de Broglie suggested that the electron, then regarded as a purely material corpuscle, also exhibited wave properties in the way the photon, the particle of light, does. All subatomic particles have subsequently been shown to behave both like waves and corpuscles. Thus, the photon, but also the electron or proton that are matter particles, travel like waves, producing interference patterns, yet interact with matter as point-like corpuscles. This phenomenon, known as wave-particle duality, still remains ill understood, as this duality has no counterpart in classical physics, whose objects of study are either waves or corpuscles, but not both simultaneously (studies by Couderc and colleagues constitute exceptions: they showed that macroscopic oil droplets on a vibrating fluid bath could exhibit behaviors analogous to quantum particles, like interference [61] or orbit quantization [62]). Ultimately, the specific behavior of a particle depends on the kind of measurement being performed, as affirmed by Bohr’s complementarity principle [2], and therefore on the apparatus [42]. The nature of physical reality is hazy, as outcomes of experiments are *unpredictable*, and as Heisenberg’s *uncertainty principle* [38] asserts that the values of noncommuting observables cannot be known simultaneously with exactitude. Pauli’s *exclusion principle* [69] further asserts that electrons, which are defined by four quantum numbers within the atom, can never present the same exact set of quantum numbers, resulting in different spatial probabilities and making their positions mutually exclusive. This principle would be responsible for the fact that material bodies do not mingle and for our sensation that matter is solid, even though atoms would be mostly made of vacuum.

The Copenhagen interpretation assumes that observables exist in superposed states, i.e., that they lie in a combination of all possible states simultaneously and remain in this indefinite condition until a specific event, known as the *collapse of the wavefunction*, actually takes place. This collapse arises whenever a measurement is performed onto the particle, forcing it to settle into one of its allowed eigenstates. A quantum system can only take on specific values upon measurement, and this property is commonly known as *quantization*. The fact that, upon measurement, the particle loses its condition of superposed state and always ends up in one of its eigenstates has currently no satisfactory explanation and constitutes the *measurement problem* [27]. The particle will then remain in that same state upon subsequent measurements. *Superposition*, quantization, wavefunction collapse, and measurement problem have no equivalence in classical physics.

**Figure 1:**
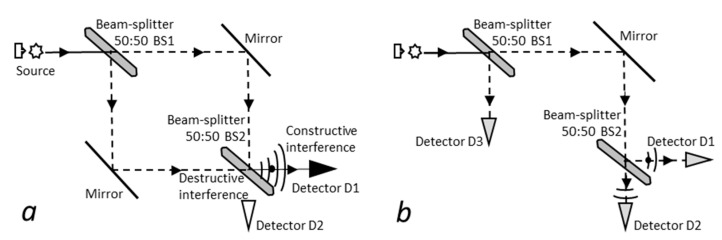
Mach–Zehnder interferometer experiment. (**a**) Interference occurring at beam-splitter BS2 causes particles to be detected only at detector D1. (**b**) Particles undetected by detector D3 do not undergo interference at BS2 and are detected by both detectors D1 and D2.

Another specific feature of quantum systems is that they seem to follow all possible routes simultaneously [70]. In a Mach–Zenhder interferometer (Figure 1a) [71] for instance, the only possible way to account for observations is to consider that a single particle goes *through all possible paths simultaneously* and subsequently interferes with itself [72] to reach only detector D1. If a detector is then placed on one of the two routes, it will detect the particle half the time, which is expected, but this setup will also affect the trajectories of undetected particles that went the other way, as they are now detected in both detectors D1 and D2 (Figure 1b, see Section 4.4). It is as if the measuring apparatus, or the *will to measure*, or the *consciousness* of the observer even [23], had changed the fate of particles that followed the other route, challenging the principle of objectivity. These properties certainly have no counterparts in classical physics.

Another peculiar quantum phenomenon is entanglement, first discussed in the famous paper by Einstein, Podoslski and Rosen [7]. Entangled particles must have been in contact prior to being sent far apart in different directions, and having their states recorded independently. Bell’s theorem [28] provides testable inequalities that allow discriminating between two different conceptions of particles, i.e., those with predetermined properties on the one hand, and those with undetermined properties on the other hand, exhibiting distinct correlation rates. Experiments yielded the correlation rates predicted by quantum theory. This phenomenon has been evidenced in many conclusive experiments since those conducted by Aspect and colleagues [29], and the last loopholes have been closed [31,73]. For the Copenhagen interpretation, two entangled particles constitute a single non-separable entity, no matter how far apart they stand, thus rejecting locality. A measurement performed onto one entity could also be instantaneously communicated to the other through some unknown potentially superluminal signal, thus contradicting special relativity.

Altogether, these observations and associated worldview led to the rejection of the classical principles of *causality, objectivity, locality, realism and determinism*. However, despite the accurate predictions of quantum mechanics, a minority of realist physicists, including some founders of quantum theory such as Einstein, Schrödinger, or de Broglie, notoriously disagreed with its standard interpretation. They believed that the observed stochasticity in measurements [74,75] was not due to an intrinsic randomness of nature, but rather to an incompleteness of the theory [7,76]: does the wavefunction completely represent the quantum object [77], as quantum mechanics advocates? Born also notably disliked the strictly positivistic standpoint saying it is meaningless to ask what underlies waves and particles [8]. He argued that it is legitimate to infer the existence of a bullet when a man fires a gun and another collapses, even if the bullet cannot be seen [6]. While accepting its predictive framework, realists regarded quantum theory as an approximate description of a deeper, more complex reality that would involve underlying processes. In their view, quantum mechanics would emerge from a hidden variable theory describing that deeper reality.

## 3. Electrodynamical Electron Model Involves Numerous Fluctuating Subparticles

**Figure 2:**
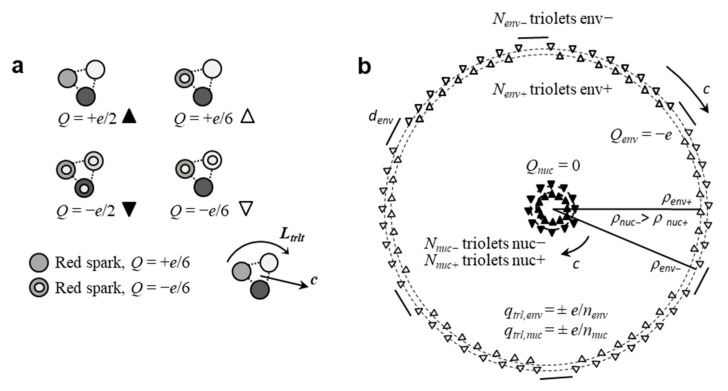
(**a**) Colorless triolets made of three colored (±*e*/6) sparks exhibit four different electric charges, represented by filled or hollow upward or downward triangles. (**b**) The granular model of the electron at rest presents triolets revolving at light velocity and constituting an envelope and nucleus.

In a causal and objective relativistic electrodynamical model [57], the electron at rest is considered as a dynamical system involving numerous subparticles called triolets, which travel at light velocity and exhibit some intrinsic angular momentum. Triolets would possess electric charges (±*e*/6) or (±*e*/2) and would be made of three colored (±*e*/6) corpuscles denoted sparks [78], bound beforehand by the strong interaction (Figure 2a). Sparks were conjectured so as to make a subatomic chemical theory possible. Triolets would be colorless and only submitted to electromagnetic and centrifugal forces. They would revolve along different coplanar circular orbits depending on their electric charge, forming an envelope and nucleus (Figure 2b), and would exhibit oscillatory microcurrents reminiscent of Schrödinger’s Zitterbewegung model [79].

This granular model is based on natural interpretations of observables, as it interprets (*i*) spin as being the sum of angular momenta of envelope triolets, (*ii*) the classical and anomalous magnetic moments as being generated by charged triolets revolving at the envelope and nucleus respectively, (*iii*) electron mass as being a manifestation of its total cohesion electromagnetic energy. Notably, both the envelope and nucleus exhibit kinetic energy [66], negative cohesion potential energy (typical of bound systems), satisfy the *Virial theorem* [80], and provide natural interpretations for *Planck’s* and *fine-structure constants*.

In this model, the globally neutral nucleus presents perfectly intertwined positive and negative triolets, conferring a tight symmetrical substructure. The envelope in contrast is negatively charged and contains more negative triolets than positive ones. Hence, positive and negative envelope triolets would be imperfectly intertwined, presumably inducing a certain instability, leading to radial or transverse fluctuations. Conceivably, several stable wave states could exist, much like modes in a vibrating rope. Importantly, the model was found to be coherent only if the envelope possessed (±*e*/6) triolets and the nucleus (±*e*/2) triolets.

The envelope would allegedly guide the nucleus [8,47,48], sense the envelopes of other particles through their electromagnetic field, and exhibit wave behavior. The instability would generate a complex (possibly chaotic) dynamics [20,54]. This worldview shares features with hydrodynamical interpretations of Schrödinger’s equation [53,55], and with the de Broglie–Bohm pilot-wave interpretation of quantum mechanics [49]. Note that in the granular model, the envelope wave is part of the particle itself, instead of being external to it. Note that the nucleus may be the seat of another wave, reminiscent of de Broglie’s double solution theory [51,52]. Notably also, the model seems compatible with both conclusions of Myrvold’s study [81] on wavefunction status, namely that wavefunctions are built upon “configuration spaces that are not fundamental, but rather are derivative of structures defined on ordinary spacetime”, and second, that “the value assigned to a point in configuration space […] depends on the global state”.

Using Liénard–Wichert potentials from (non-quantum) relativistic electrodynamics [67] to account for the electron self-interaction [66], electron mass and muon mass were derived from the stability of the model’s substructure. The study thus implemented Lorentz’ hypothesis advocating the electromagnetic origin of mass from an objective criterion instead of an arbitrary parameter. The number of triolets was iterated until the various constraints (charge, spin, angular momenta, stability, etc.) were satisfied, yielding a solution that involved 126 (±*e*/6) triolets at the envelope and 18 (±*e*/2) triolets at the nucleus and predicted electron mass. Altogether, the model illustrates the possibility of constructing causal, objective, local and realist models of subatomic particles beneath the Compton scale.

## 4. Realist Interpretation of Quantum Phenomena, Properties, and Principles

Most emblematic properties and principles of quantum theory are interpreted here in the light of the granular electron model, with the aim of sketching a coherent and fundamentally realist interpretation of quantum mechanics, by which particles and their trajectories would become intelligible in geometrical terms and every quantum property would fit naturally into a unified interpretation [66].

A realist interpretation of some of the most abstract concepts of quantum mechanics is sought here in terms of subcorpuscles possessing well-defined positions and velocities. An objective solution requires that perturbations due to measurements be replaced by objective criteria, possibly arising at the level of subparticles. The solution proposed here allegedly allow accounting for most emblematic experiments, properties, and principles associated with quantum theory, constituting a causal, objective, local, fundamentally realist, albeit contextual and unpredictable granular interpretation of quantum theory.

### 4.1. Wave-Corpuscle Duality, Probability Densities, and Physical Reality

All particles are found to interact as point-like corpuscles in scattering experiments [82] or to behave as extensible waves [83]. This phenomenon, considered to be fundamental by Bohr, is called wave-particle duality, and “both the wave and the particle aspects of matter have objective significance” [42]. It seems particles cannot display both kinds of behaviors simultaneously, constituting the complementarity principle [2]. Note that this principle may be only apparent as experiments by Ghose and Roy [84] and Afshar et al. [85] seem to show that both wave-like and corpuscular behaviors can be observed simultaneously. Heisenberg [12] therefore believed that particles could not be apprehended by the human mind and that only abstract representations were possible ultimately. Yet, for realist physicists, natural phenomena must violate complementarity [10].

Although we have some intuition of what corpuscles are (i.e., some kind of small, possibly hard, round-shaped elements), the concept of a wave in *vacuum* is less straightforward. In theories prior to quantum mechanics, waves constituted high-level descriptions of the coordinated undulation of numerous underlying molecules. How would a wave be an adequate description of a single particle? Intuitively, this may suggest that particles could be made of numerous fluctuating subparticles, and the quantum mechanical wave would emerge from the collective movement of their sub-elements.

Moreover, as there are two different behaviors, particles could possibly be made of two different substances: one that would behave as a wave, and the other interact in a point-like corpuscular manner. In the granular electron model, the envelope is made of ±*e*/6 triolets, while the nucleus is composed of ±*e*/2 triolets. Thus, envelope triolets could sense the electromagnetic fields exerted by the envelopes of other particles and guide the nucleus, while nucleus triolets would interact in a corpuscular way. The electron itself would not need to be small anymore, as corpuscular interactions could effectively reduce to contact interactions among ±*e*/2 triolets, which are presumably tiny subparticles themselves. These conjectures constitute objective propositions that could explain both kinds of behaviors.

Contrary to de Broglie–Bohm theory or Selleri’s ghost-like non-energetic waves [86], in the granular model the wave is incarnated by the envelope that is not distinct from the particle, but part of it rather. Bohr’s complementarity principle is violated by the model as both aspects simultaneously exist, albeit at different levels. Both aspects would be incarnated by two kinds of triolets, themselves ultimately made of three sparks conceived as ±*e*/6 colored subcorpuscles [78] (Figure 2a). Hence, wave-corpuscle duality would be only apparent and would correspond to a duality in function, not in substance.

Unlike *monist* conceptions in which ‘everything is waves’ [87,88,89,90,91], and although it is distinct from other corpuscular conceptions in which “everything is corpuscles” [70,92,93], our worldview unifies both aspects under a single corpuscular reality, but remains *dualist* nevertheless as the existence of independent electromagnetic fields is required alongside particles [10,26] (in the granular model, electromagnetic fields exist in vacuum without carrier particles [57]).

Heisenberg’s principle [94] can also be reinterpreted in light of this granular model. Einstein regarded this principle as suggesting that particles were extended: “It seems to me certain that we have to give up the notion of an absolute localization of the particles in a theoretical model. This seems to me the correct theoretical interpretation of Heisenberg’s indeterminacy relation” [95]. Considering Schrödinger’s Zitterbewegung electron model [79] (i.e., a loop of current whose radius is reduced Compton wavelength *ħ/mc*, where *ħ* is reduced Planck constant, *m* the electron mass, and *c* light velocity), Hestenes [66] argued that if *Δx* and *Δp_x_* respectively represented the uncertainties in width and momentum, with *Δx* equated to *ħ/mc* and *Δp_x_* to *mc*/2 (corresponding to kinetic energy *mc*^2^/2), then Heisenberg’s uncertainty relation *ΔxΔp_x_* = *ħ*/2 would be directly deduced. Accordingly, Jabs [96] considered that subatomic particles had no sharp position or momentum, and that the ranges *Δx* and *Δp_x_* stemmed from properties of the associated wavepacket. Remarkably, in the granular electron model, the wavepacket may be conceived as an extended territory of radius *ħ/mc* containing all envelope subparticles, and envelope kinetic energy is precisely *mc*^2^/2 [57]. Note that both the nucleus and envelope are real and energetic [97] in the granular model, in agreement with Pusey et al.’s theorem [98], according to which unreal quantum states cannot reproduce the predictions of quantum theory. Noteworthy, Heisenberg’s original formulation of the uncertainty principle considered a particle with a definite but unknown trajectory, that would be subject to unpredictable and uncontrollable disturbance [94].

**Figure 3:**
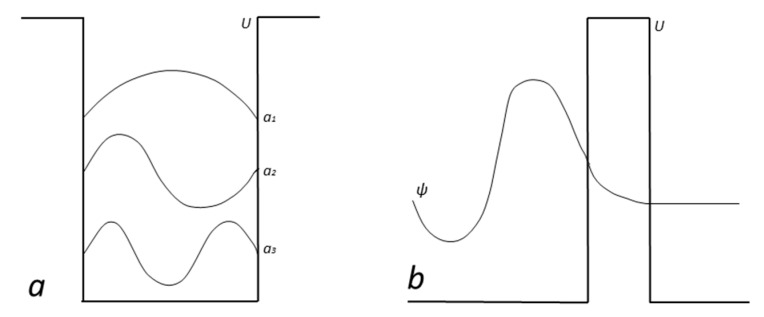
(**a**) Eigenstates in one-dimensional potential well. (**b**) Tunneling.

In quantum mechanics, quantum states have been related to standard probability distributions [99] and *probability densities* allow determining the probability of finding the (point-like) particle at any specific position in the atom upon measurement. However, the subparticles of a granular electron could actually be spreading over an extended atomic territory. Probability densities could then describe the territories occupied by subparticles on average. Accordingly, the *one-dimensional potential well* [5] (Figure 3a) could make the subparticles composing the electron follow complex dynamics over extended territories until reaching a state of equilibrium in which their trajectories stabilize. Note that its eigenstates effectively resemble the modes of a vibrating rope. Thus, wavefunction |*ψ*> could be related to the distribution of subparticles.

*Tunneling* [5] may be similarly interpreted. Conceivably, envelope triolets would be able to penetrate to a certain extent inside the atoms constituting the potential barrier. They would then have non-zero probability (Figure 3b) of guiding the electron nucleus through the barrier, allowing the particle to escape.

Likewise, Pauli’s principle could express the fact that electron subparticles in the atom would occupy *exclusive territories*. Those territories would electromagnetically repel each other and organize themselves so as to form stable resonances [66], even though they would remain attracted by the stronger nuclear electric charge. To our knowledge, this constitutes a novel realist (in the geometrical sense) interpretation of Pauli’s principle. In the quantum view, the atom is made of a tiny nucleus and point-like electrons surrounding it, making the atom in particular, and matter in general, appear as almost entirely made of vacuum. In contrast, in our picture, electrons are not point-like but extended and composed of numerous subparticles occupying territories roughly the size of the Compton scale. Hence the atom (and matter) could actually be *filled* with numerous subparticles constituting exclusive territories, repelling each other electromagnetically, and thus possibly being responsible for the *hardness of matter*. In this view, physical reality would become *concrete* again.

### 4.2. Dynamical Systems, Stable States, and Quantization

Quantum systems do not yield arbitrary results upon measurement; rather, their final states are quantized: measurements force the system to settle into one of their eigenstates, and the values taken by their observables will depend on that state.

States of quantum systems are actually wave states, which may correspond in the granular electron model to states of the envelope, which is composed of positively and negatively charged subparticles. The fact that the envelope bears the full electron charge implies the existence of adjacent negative subparticles that repel each other and complicate the overall envelope dynamics and stability. Therefore, envelope subparticles could undergo complex (possibly periodic or chaotic) dynamics [54] involving radial or transverse fluctuations, constituting various states of the envelope. In general, the envelope would be in unstable states, but fluctuations could stabilize and converge towards stable states, like modes of a vibrating rope. Once in a stable state, the envelope would remain in that state upon subsequent measurements. Stable states could correspond to the eigenstates of quantum mechanics, and the envelope would thus exhibit quantization.

**Figure 4:**
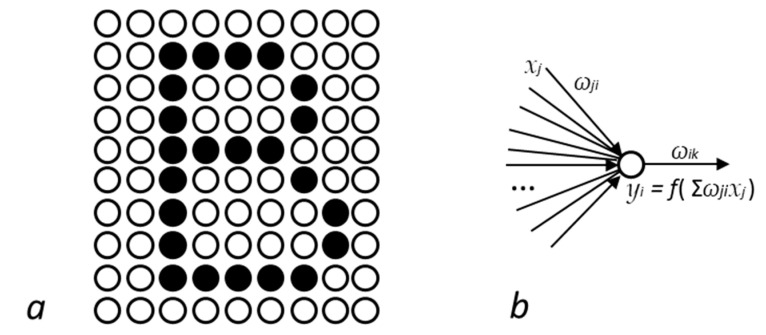
(**a**) Pattern (attractor) memorized in a recurrent neural network. (**b**) Each neuron receives the weighted signal from all other neurons and emits a signal accordingly.

Drawing inspiration from ‘t Hooft’s cellular automaton interpretation [100], could recurrent neural networks help apprehend quantum mechanics? The existence of convergent stable states (or attractors corresponding to global or local energy minima) is a general property of dynamical systems [59]. *Recurrent Neural Networks* [58], which are particular dynamical systems belonging to the field of Deep Learning in Artificial Intelligence, notably share this property. The so-called *Boltzmann machine* [101] and *Hopfield network* [102] are examples of recurrent neural networks adapted from physics. These neural networks (Figure 4a) are constituted of *N* fully interconnected *artificial neurons* (Figure 4b), which are mathematical abstractions of brain neurons. In recurrent networks, each artificial neuron *i* receives a signal from all other neurons *j*. Each signal is multiplied by a *synaptic weight ω_ij_*, specific to the connection (or synapse) between the two neurons. Synaptic weights can be positive or negative, corresponding to correlated or anticorrelated neurons respectively, and altogether constitute the memory of the system. Several convergent *patterns* of excited neurons, called attractors, can be memorized (Figure 4a). Attractors can be represented by vectors of *N* excitation states 0 or 1, hereby denoted |*a_n_*>, designating the *n*^th^ attractor of the network. During the learning phase, synaptic weights are adjusted so as to make the whole system learn, memorize, count or dream [58].

Once the network has been trained, a ‘question’ |*ψ*> may be submitted to the network by assigning a signal value *x_i_* (0 or 1) to every neuron *i*. The recurrent network will then enter a dynamical process, in which every neuron *i* receives the weighted signal (*ω_ij_x_j_*) from every incoming synapse and triggers a signal accordingly. In turn, this signal will be sent to all neurons connected through the weighted synapses. The network will reiterate this process until it reaches a stable state, i.e., an attractor |*a_n_*> of the system, which exhibits constant signal value for every neuron. The system can then be seen as having inferred answer |*a_n_*> from question |*ψ*>. Recurrent networks will often, but not necessarily, converge towards the attractor nearest to the question. Such systems are commonly used to recognize images from blurred inputs, such as hand-written post-codes on mail envelopes for instance.

The resemblance to quantum mechanics is straightforward. Recurrent networks are somewhat quantized, as their dynamics always converges towards one of their attractors. During the dynamical process, before reaching an attractor, their state is undefined. It is evolving somewhere in between the various attractors, and it is unclear which one will be reached eventually. Once in an attractor, the system will remain in that state. Thus, a recurrent neural network exhibits many features of quantum mechanics. Conversely, could a quantum system be conceived as some kind of dynamical system converging towards attractors? It is tempting to regard quantized particles as collections of interacting subparticles (as in the granular model) undergoing complex internal dynamical processes converging towards attractors. Accordingly, wavefunction collapse would then correspond to unstable states converging towards one of the system’s stable states. Thus, quantization would reflect the existence of a finite set of attractors.

The current state of a dynamical system would not generally be in a *superposition* of attractors, unless the set of eigenstates forms a basis in vector space, allowing any state, at any time step of the dynamical process, to be written as a linear combination of eigenstates [5]. Noteworthy, the modes of a vibrating rope may be used to approximate any rope undulation in the same way Fourier series can approximate any signal [80]. Thus, the superposition principle would possibly only convey an approximation akin to Fourier series.

In the granular model, envelopes would conceivably be capable of storing in parallel several independent states, presumably using different wave features (longitudinal or transverse frequencies, amplitudes, etc.). This ability could be at the basis of commuting observables, while *noncommuting observables* would mobilize the same wave features. Thus, the measurement of an observable would alter its corresponding wave features, and thus possibly the values of all noncommuting observables sharing those particular features.

### 4.3. Collapse of the Wave-Packet, Measurement Problem, and Causality

A particle (generally in a superposed state) settles into an eigenstate upon measurement. What exactly triggers that reaction known as the collapse of the wave packet? The collapse should be conceived as a process distinct from the act of measurement [20]. It arises every time a measurement is performed, suggesting a causal rather than stochastic relationship [19]. In which sense is it fundamentally different from the undisturbed deterministic evolution described by Schrodinger’s linear equation? What physically distinguishes measurements from other interactions? Could objective criteria triggering wavefunction collapse be proposed? Why should a measurement always result in the particle reaching an eigenstate instead of remaining in a superposed state? Could this measurement problem [27], which has presently no satisfactory explanation, be interpreted in realist terms? Some conjectures are proposed here.

The act of measurement may be effectively incarnated by interactions occurring between subparticles constituting both the observed system and measuring device, or apparatus. In quantum mechanics, we speak of *contextuality* to mean that values of observables depend on the measurement context [75], which includes the dependence on the apparatus and the order in which measurements are made.

Consider what happens in metals that are heated and then slowly cooled down so as to form a purer state. Heat effectively brings in noisy energy in the form of random vibrations, allowing atoms and electrons in metals to escape their local minimum energy states [103]. The slow cooling of metals then allows atoms and electrons to settle progressively in ever lower energy states. Eventually, metals will become more homogeneous and present fewer impurities.

Likewise, the act of measurement may be seen as bringing noisy energy to the system due to vibrations of apparatus subparticles. The interactions between system and apparatus subparticles could disturb both and make them converge towards states of lower global energy or higher entropy, presumably corresponding to eigenstates. Envelope wave-states would reach resonance with the oscillations of apparatus subparticles: after measurement, the detector state is correlated with the measured system state [4,18]. This complex process would not be described by the linear evolution of Schrödinger’s equation; rather, particles would appear to *jump* [104] from superposed states to quantized eigenstates, but the whole process could remain entirely causal at the substructure level. Conceivably, subparticles could be submitted to causal electrodynamical interactions, as in the granular model, while their trajectories would remain well-defined. In this view, the probabilistic nature of quantum mechanics would be only apparent.

The involvement of apparatus subparticles would constitute an objective criterion that allows distinguishing between the two possible evolutions of quantum systems. By relying on a disturbance criterion instead of the stochastic collapse theory of Ghirardi et al. [19] for instance, this proposal presents an objective, causal and local interpretation of von Neumann’s collapse of the wave-packet, and a possible solution to the measurement problem.

### 4.4. Simultaneous Multipath Exploration, Particle Detection, and Objectivity

The quantum property of simultaneous exploration of all possible paths is perhaps best illustrated in the Mach–Zehnder interferometer experiment [71]. Particles are emitted individually and first encounter a beam-splitter BS1 allowing the particle to follow two distinct possible paths before reaching a second beam-splitter BS2 (Figure 1a). Either way, the particle is detected by detector D2. For a single particle, this observation only makes sense if the wave associated to the particle has travelled through both paths simultaneously and has interfered with itself in BS2 [72], so that constructive interference forces corpuscular detection in D2, while destructive interference prevents detection in detector D1.

Interestingly, the experiment also challenges the principle of objectivity. A detector D3 placed on one path (Figure 1b) will detect the particle 50% of the time (this is expected), but particles going the other way will also be disrupted: they will then be detected half the time in each detector D1 and D2. It is as if the act of *non-measurement* on the first path, or even just the will to measure [23], were sufficient to change experiment outcome. The two-slits experiment also exhibits this peculiar property [105]. Such experiments show how particle behavior can be affected by measurement and illustrate why objectivity seems to be unverified in quantum mechanics.

In the granular model, the electron is composed of a tightly bound nucleus made of ±*e*/2 triolets and a loosely bound envelope made of ±*e*/6 triolets. Envelope triolets are conceivably loose enough to be separated when passing through beam splitters. Hence, envelope triolets would explore both ways, while the tight nucleus would follow only one path [106]. Noteworthy, this kind of objective solution is also compatible [107] with phenomena observed in delayed-choice experiments [108,109], wherein the setup is determined at last picosecond.

In our view, the particle envelope would be separated and explore all routes. In the granular model, the envelope fraction remaining with the nucleus would act as a guiding wave, as in the de Broglie–Bohm interpretation. When reunited, both envelope fractions would interfere with each other at beam-splitter BS2, guiding the nucleus along the constructive interference pathway [107]. In case a supplementary detector is placed in D3, the undetected empty envelope fraction would be physically blocked in D3, preventing the occurrence of interference at BS2, thus explaining why the nucleus is then detected in both detectors D1 and D2.

Conceivably, detectors would not detect the envelope, only the nucleus, possibly because ±*e*/2 triolets would be the only triolets interacting directly with detectors (the particle nucleus might possibly exchange triolets with other particle nuclei, changing particle identities, in contrast to envelope interactions involving only wave state alterations). Accordingly, the envelope fraction without nucleus would not trigger detection. These conjectures constitute possible objective propositions for particle detection.

Note that an alternative possible explanation involves the propagation of ghost-like (particle-free) electromagnetic waves in vacuum [86]. In this view, the envelope would keep its integrity and remain attached to the nucleus, while electromagnetic particle-free waves would propagate along all possible paths in vacuum.

Hence, accounting for the quantum simultaneous multipath exploration is possible within a local objective framework. In principle, particle detection and the subjective role of the observer could be replaced by objective reactions [22], preserving the principle of objectivity.

### 4.5. Unpredictability, Hidden Variables, and Determinism

Since quantum theory seems incompatible with the macroscopic world [16] and since a satisfactory description for wavefunction collapse is allegedly still lacking, quantum theory would not be complete [42,110], and an underlying theory involving hidden variables may still be lacking [56]. Bell wrote notably: “Either the wavefunction, as given by Schrödinger’s equation, is not everything, or it is not right” [77].

Various impossibility proofs developed to prohibit hidden variable theories allegedly made excessive assumptions regarding their presupposed properties [111]. Of note, Bell did not believe that impossibility proofs excluded the possibility of a deeper level of description (“What is proved by impossibility proofs is lack of imagination” [112]). Rather, he viewed them as identifying conditions constraining acceptable solutions [111]. “Local realism has been equated with deterministic, local, noncontextual hidden variables”, which is too restrictive [15]. Accounting for impossibility proofs [18,39,40,41], what kinds of hidden variable theories would be acceptable? Bell’s inequalities [28] are based on a factorizability condition, which implies noncontextual hidden variables with Kolmogorovian structure [15]. However, this factorizability condition is only an assumption. It has been shown that the observed violation of Bell’s inequalities could be due to contextuality [32,33,34] and non Kolmogorovian probabilities [36,37]. Noncontextual hidden variable theories are also incompatible with quantum mechanics [33] and ruled out by Gleeson and Kochen-Specker theorems [75]. Incidentally, Gudder [32] provided a proof of the existence of a contextual hidden variable theory agreeing with the statistical predictions of quantum mechanics [75]. Alternatively, a theorem by Masanes et al. [113] suggested that either *predictability* or *signal locality* (i.e., the impossibility to send signals faster than light [35]), which are operational properties, could be dismissed instead of noncontextuality or locality [35]. As superluminal velocities have never been recorded, predictability could be discarded, while signal locality would be conserved.

Since “the result of an observation may reasonably depend […] on the disposition of the apparatus” [40] and since “measured probabilities of different outcomes depend strongly on experimental context” [114], contextuality would be plausible and may remain compatible with causality, objectivity, and locality within a fundamentally realist worldview. Contextuality could mean that measurement outcomes depend upon hidden variables in the apparatus [115]. Similarly, unpredictability would also be acceptable, as it seems plausible that numerous vibrating subparticles belonging to the incoming particle (as in the granular model) and interacting with those of the apparatus would yield unpredictable outcomes.

The positions and momenta of envelope subparticles would constitute possible hidden variables. Even within a causal framework, the *lack of knowledge* about the positions and momenta of subparticles would naturally impede making predictions. The situation is similar to that of statistical mechanics, in which the high number of possible configurations implies that predictions should only be treated statistically [103]. This might indeed be the reason why quantum mechanics, as a probabilistic theory, is so successful. However, this should not be taken to imply that the laws governing particles are intrinsically non-causal, non-objective, non-local or non-realist, or that the underlying level is beyond scrutiny.

Particles could be highly sensitive to their environment and generally undergo underlying chaotic dynamics that would prevent high-level observables from possessing determined values. If the existence of contextuality and unpredictability are ultimately established, then *high-level determinism* (i.e., particles possess definite high-level properties that determine experiment outcomes) should be abandoned.

### 4.6. Entanglement, Memory Imprinting, and Locality

Two particles are entangled if their states are related so that a measurement performed onto either one of them determines instantly the result of a similar measurement subsequently performed on the other, no matter how far apart they stand from each other. To be entangled, particles must have previously interacted. There are effectively three possibilities: (*i*) their states were entangled from the start; (*ii*) they form a single non-separable entangled system; or (*iii*) a signal is sent from the measured particle to the other. Solution (*i*) means that both particles carry from the start a specific property that would somehow decide future outcomes of measurements (Mermin notably wrote: “in the absence of spooky actions at a distance, it is hard to understand how this can happen unless the earlier measurements are simply revealing properties of the subsequently measured particle that already exist prior to their measurement” [111]). Solution (*ii*) is the one adopted by quantum mechanics, which treats entangled particles as a single non-separable system. Thus, measurements performed onto one of the particles would automatically force the other particle to jump into the correlated eigenstate, even if they were previously in indefinite states. Note that *non-separability* implies the rejection of locality. To test solution (*iii*), experiments were prepared so as to necessitate signals travelling at superluminal velocity in order to reach the other particle before it is measured. This solution is therefore incompatible with special relativity. Noteworthy, such velocities have never been observed [15]. Only solution (*i*) is compatible with locality.

Testable criteria were needed to decide among these three possibilities. Bell’s inequalities [28] allow testing the validity of local realist theories. Bell hoped the latter would be comforted, and the Copenhagen interpretation rejected. Bell’s inequalities were later refined to ease testability [116,117]. Experiments were designed and performed, e.g., [29,30,31]. Correlations were recorded, ruling out both purely stochastic solutions [114,118] and predictable solutions [1,35].

An intermediary solution between *irreducible randomness* and *counterfactual definiteness* is conceivable: not all variables need be definite to convey correlated states. In agreement with type (i) solutions, Kupczynski [114,118] proposed that wave-packets would carry *partial memory* of the initial interaction, so as to make entanglement possible. In the granular model, the electron envelope, because of its complexity, would possibly be capable of imprinting the *past history of the particle*, i.e., its creation and encounters with particles and fields. The partial memory could be stored within some characteristics of the envelope dynamical wave states (e.g., longitudinal or transverse frequencies or amplitudes in the granular model). Two entangled particles would then travel from the start with their states correlated [119] and would naturally exhibit correlations upon measurement. Two different levels of realisms are conceivable: *strong realism*, whereby everything is determined (this is rejected by experiments), and *fundamental realism*, whereby only low-level properties would be definite, allowing the conservation of correlated wave-states through memory imprinting. Entangled particles would possess somewhat similar or symmetrical wave-states storing partial memory within the values of low-level hidden variables (e.g., positions and momenta of subparticles).

Experiments “neither observe strict correlations nor anticorrelations” [25]. Besides, two indefinite quantum systems should not be correlated [118]. The envelope states of entangled particles would not generally be determined, evolving in unstable states, presumably following Schrödinger’s equation but remaining extremely sensitive to perturbations or interactions with the apparatus. However, they could still share enough common history through imprinting to exhibit correlations. Interestingly, the measured correlation rates can also be predicted within local theories [65,120]. Contrary to what Salart et al. advocate [30], locality could be preserved if contextuality is established [121], and nonseparability or superluminal signals would not be necessary.

## 5. Conclusions and Perspectives

According to Bell, “subjectivity and indeterminism are not forced on us by experimental facts but are a deliberate choice” [112]. In this study, the possible existence, at least in principle, of a fundamentally realist interpretation of quantum mechanics is proposed. Drawing inspiration from a relativistic electrodynamical model of the electron constituted of numerous fluctuating subparticles [57], and more generally from non-linear dynamical systems, a granular substructure forming an envelope and nucleus was suggested for all subatomic particles. Causal, objective, local, fundamentally realist, albeit contextual and unpredictable interpretations were presented for several unresolved issues of quantum mechanics. Specifically, objective criteria (the possible existence of two kinds of subparticles, of dynamical systems converging towards stable states, of memory imprinting, etc.) were proposed to provide novel interpretations for emblematic quantum phenomena, properties, or principles (wave-particle duality, quantization, superposition, apparent loss of objectivity, exploration of all possible paths, collapse of the wavepacket, measurement problem, unpredictability, entanglement, etc.), hinting at a “quantum mechanics without observers” [22]. Accounting for constraints from impossibility proofs, original hypotheses were conjectured:

- Particles would be composed of subcorpuscles organized into an envelope and nucleus exhibiting wave-like and corpuscular behaviors respectively.
- All entities would ultimately be corpuscular, and wave properties would emerge from the undulation of the numerous corpuscles composing the envelope.
- The envelope would generally undergo unstable non-linear dynamics, but stable states would exist, much like modes for a vibrating rope; the eigenstates of quantum mechanics would correspond to those stable states.
- The envelope would guide the nucleus as in the de Broglie-Bohm pilot-wave interpretation.
- The act of measurement would force the envelope to converge and stabilize into an eigenstate (collapse of wavepacket), possibly because of interactions between system and apparatus subparticles.
- Measurements would alter the envelope wave-state, perturbing the values of other dependent (noncommuting) observables.
- Particle detection would involve direct interaction between nucleus subparticles of system and apparatus particles.
- Partial particle history (i.e., its creation and encounters with other particles and fields) would be imprinted within the envelope dynamical wave-state;
- Entangled particles would have characteristics imprinted from the start, thus exhibiting correlations and preserving locality.
- Positions and momenta of subparticles would be determined, while higher-level observables would not generally.
- High-level indeterminism and contextuality (i.e., unknown positions and momenta of system and apparatus subparticles) would cause the unpredictability of measurement outcomes.
- Quantum mechanics would constitute a high-level wave-like description of underlying causal, objective, local and realist processes, and would not be probabilistic in essence.
- Probability densitieswould describe average territories occupied by the subcorpuscles composing the extended particles.
- Matter, which seems almost empty in the quantum mechanical picture, would appear full of numerous fluctuating subparticles, constituting exclusive territories in the atom (Pauli’s principle).

**Figure 5:**
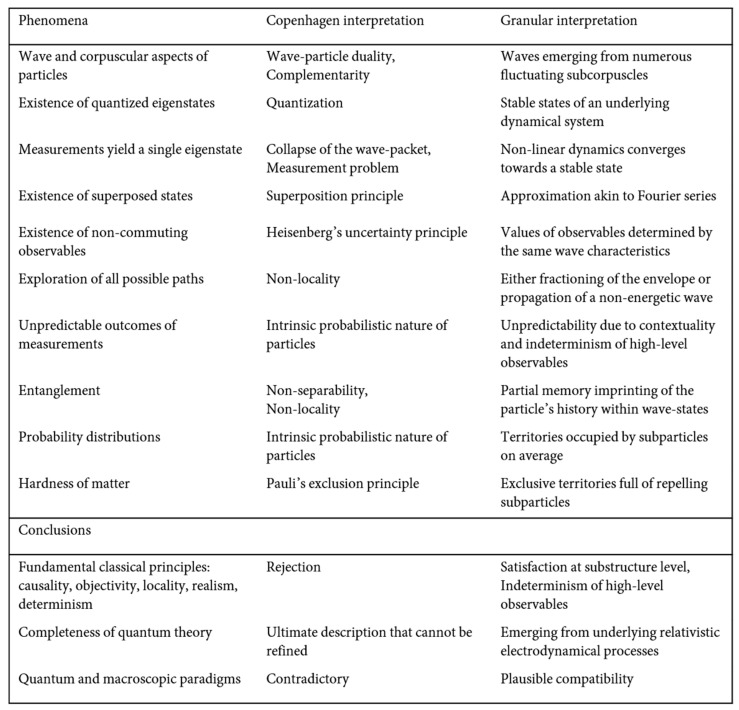
Comparison between the Copenhagen and granular interpretations of quantum mechanics.

The proposed worldview involves a deeper level of description. Quantum mechanics would not be the ultimate description of nature beneath Compton scale but would emerge as a high-level description of wave phenomena incarnated by the fluctuations of subparticles: the wavefunction would correspond to a real wave, in agreement with the theorem by Pusey et al. [98]. Comparison between the Copenhagen and granular interpretations of quantum mechanics is provided in Figure 5. Future philosophical studies should refine the present interpretation and account for phenomena and properties not included in the present study. Experiments should be devised and carried out in order to validate or invalidate the proposed conjectures. In particular, experiments challenging the orthodox quantum worldview, e.g., [84,85,122,123], should be re-examined.

The present interpretation should be supplemented with mathematical studies demonstrating that it is compatible with the uncontested predictions of quantum theory. It is one thing to say that the granular model could exhibit wave-like behavior and stable states, and quite another to demonstrate mathematically that it is indeed the case. Could a wave equation describing the dynamics of envelope subparticles be determined? Could it prove compatible with Schrödinger’s or Dirac’s equations? Could eigenstates be geometrically represented by actual positions and momenta of subparticles? Could the collapse of the wavefunction and convergence towards eigenstates upon measurement be mathematically demonstrated? A whole program of mathematical modelling, relating granular models to various properties of quantum theory, is therefore necessary to verify the soundness of our propositions.

Additionally, could granular models be constructed for subatomic particles other than the electron? Could models of non-elementary particles, such as hadrons, be constructed by assembling several granular quark models? Would this worldview also apply to more complex constructions, such as the atom, molecular bonding, or molecules? This prospect would constitute a novel *structural subatomic chemistry*, akin to structural molecular chemistry [124], only applying to objects belonging to the world of subatomic particles. Incidentally, the numerous subcorpuscles involved in our worldview form *train-waves* (interestingly, Born [125] developed a thought experiment against determinism. Considering a single gas molecule as a moving elastic sphere, he reckoned that even tiny changes in its initial velocity would eventually result in large deviations in its trajectory. He concluded that such a system would never satisfy determinacy and would prevent making predictions. However, should a gas molecule be approximated as an elastic sphere? In the granular model, particles are stretches of numerous subparticles that behave as train-waves. Hence, the expected deviations due to point-like elastic interactions might never arise). Thus, our worldview encompasses both stances—‘all is waves’ [87] and ‘all is corpuscles’ [70]—albeit at different levels. Note that these train-waves, reminiscent of superstring theory [126], form loops, which are themselves reminiscent of quantum gravity loops [127,128].

It seems most fundamental principles of classical physics may be conserved beneath Compton scale with limited adjustment. This is the case of *causality* (since everything herein is governed by causal processes), of *objectivity* (since objective criteria could be proposed to replace subjectivity in particle detection, wavefunction collapse, etc.), of *locality* (since we chose to conserve it at the expense of predictability and noncontextuality), of *fundamental realism* (since underlying hidden variables would remain definite, even if high-level observables would not). Note that low-level determinism and realism are still adequate principles in our worldview, but the complexity of non-linear dynamical systems and contextuality would prevent predictability. Particles would only appear to be stochastic due to our lack of knowledge regarding underlying processes, in a manner similar to statistical mechanics [103]. Even in macroscopic physics, all phenomena cannot be described [119]. Individual predictions are often impossible for complex systems due to the high number of unknown variables, and the statistical method must be used [6]. Nature would only be more complex than previously thought [100].

How could the underlying level be causal, objective, local, realist and determinist if the emerging level, the quantum level, is not? Is this proposition not contradictory? Actually, this situation is reminiscent of statistical mechanics, in which *time irreversibility* can also emerge from time-reversible interactions because of the high number of possible configurations. Likewise, the peculiar quantum properties could emerge from underlying fundamentally realist processes.

“One is currently taught that the macroscopic and microscopic worlds are intrinsically different, the former being deterministic and the latter probabilistic” (Bush, 2010). Even though high-level observables would not be determined, our interpretation of quantum theory would be inscribed within a *fundamentally realist paradigm*, as it would involve real subcorpuscles possessing definite properties and following real trajectories in spacetime. Our interpretation would not be strictly speaking classical however, as it is founded on *relativistic* dynamics. The fact that most principles of classical physics are satisfied in our worldview would allow envisaging some compatibility between the macroscopic and microscopic worlds, suggesting the possible existence of a unified paradigm.

Altogether, a novel *granular interpretation of quantum mechanics* that conceives particles as undulating granular systems is proposed. In our view, the granular electron model illustrates the kinds of dynamical systems that could incarnate the wavefunction, while providing objective criteria to interpret quantum properties realistically. All quantum phenomena may possibly be intelligible in terms of fundamentally realist properties and dynamics. Physical reality could become concrete again, allowing geometrical representations of particle substructures and trajectories. Although other kinds of realist interpretations might be possible, we find the present interpretation elegant and insightful, as it conceives particles as systems of numerous, extremely sensitive, fluctuating subcorpuscles.

## Data Availability

Not applicable.
